# Decline in academic performance and mental health during the COVID-19 pandemic: a cross-sectional survey among Sapienza University of Rome students

**DOI:** 10.3389/fpubh.2024.1408191

**Published:** 2024-07-17

**Authors:** Leonardo Maria Siena, Ilaria Mussetto, Erika Renzi, Valentina Baccolini, Giuseppe Migliara, Antonio Sciurti, Antonio Covelli, Corrado De Vito, Carolina Marzuillo, Paolo Villari, Azzurra Massimi

**Affiliations:** ^1^Department of Public Health and Infectious Diseases, Sapienza University of Rome, Rome, Italy; ^2^Department of Life Sciences, Health and Health Professions, Link Campus University, Rome, Italy

**Keywords:** COVID-19, academic performance, mental health, university, students

## Abstract

**Introduction:**

The COVID-19 pandemic has posed significant challenges to the education system, leading to changes in student academic performance and mental health. The aim of this study was to evaluate variables relating to changes in academic performance and mental health during the pandemic.

**Methods:**

We carried out a cross-sectional study from 28 February 2022 to 13 April 2022, during the free SARS-CoV-2 screening campaign offered by Sapienza University of Rome. A structured questionnaire was constructed to explore the decline in academic performance during the COVID-19 pandemic. The Coronavirus Anxiety Scale (CAS), a validated self-reporting mental health screener of dysfunctional anxiety associated with the coronavirus crisis, was used.

**Results:**

A sample of 1,134 students was enrolled. A total of 25.4% of the participants reported a decline in academic performance. In addition, Coronavirus Anxiety Scale scores revealed that 133 (11.5%) students had a dysfunctional anxiety problem due to COVID-19. A multivariable logistic regression model showed that being a senior student (aOR: 0.70 95% CI: 0.52–0.96) and having good financial status (aOR: 0.64, 95% CI: 0.47–0.88) decrease the likelihood of a decline in academic performance, while not being Italian (aOR: 2.12, 95% CI: 1.29–3.48), having felt the need for psychological support (aOR: 2.58, 95% CI: 1.87–3.55) and being enrolled in a science/technology faculty (aOR: 1.81, 95% CI: 1.27–2.57) were more likely to result in a decline in academic performance.

**Conclusion:**

Our results show that the pandemic has affected academic performance. The COVID-19 emergency highlighted the importance of considering mental health and economic status in policymaking to effectively support students.

## Introduction

The COVID-19 pandemic has caused unprecedented disruption in education systems worldwide due to closures of schools and universities and forced changes in educational administration (1). Among the measures to prevent contagion that institutions had to implement, the transition from in-person teaching to remote or hybrid instruction was unquestionably the most impactful (2). This rapid transition, conducted with little notice and planning (2), presented numerous challenges, significantly affecting student academic performance. Research has found that students’ test scores fell and existing achievement gaps were exacerbated as a result of the shift to online learning (3); other studies have similarly reported that the transition to remote learning harmed student achievement (4). This has further underscored the need for effective intervention and support to help institutions navigate the challenges posed by the COVID-19 pandemic and ensure that students are able to continue learning and achieving their academic goals.

Beyond academic performance, many students reported increased levels of stress and anxiety during the pandemic. A survey conducted by the American Psychological Association found that more than half of all students reported feeling overwhelmed by the changes brought about by the pandemic, and nearly 40% reported feeling anxious or stressed (5). Just like the rest of the population, the impact of the pandemic has also affected various aspects of their private and social life. Economic hardships increased, both for those who relied on their families’ support and for those who were working students (6). Many were forced to suspend or leave their studies, and this contributed to the already problematic deterioration in student mental health (7, 8). Anxiety disorders, depression, and burnout syndrome are just some of the consequences of the pandemic for a considerable percentage of the population (9).

Italy, being among the first European nation to face the impact of the COVID-19 pandemic and consequently among the earliest to restructure its education system, has seen limited research on the factors influencing academic performance and student mental health during this crisis (10, 11). This cross-sectional study, carried out at Sapienza University of Rome—one of Europe’s largest universities in terms of enrollment—offers a unique opportunity to survey a large student population. The study aimed to estimate the prevalence of COVID-19 anxiety symptoms among students and assess variables linked to changes in academic performance during this challenging period. This research holds significance as it sheds light on the diverse effects of the pandemic on students and aids in pinpointing areas where interventions can be directed to strengthen both academic success and mental well-being.

## Materials and methods

### Setting and participants

This cross-sectional study was conducted during the free SARS-CoV-2 screening campaign offered by Sapienza University of Rome. The screening campaign provided a RT-PCR molecular test to the entire student population enrolled in the 2021–2022 academic year (12, 13). All students regularly enrolled were invited by e-mail to participate. While students were waiting for their turn at the screening site, they were invited to voluntarily take part in an online survey accessible via smartphone through a QR code. Whereas it was possible to take more than one PCR test throughout the screening campaign, the questionnaire was available only once. The survey was conducted from 28 February 2022 to 13 April 2022. The study was performed in accordance with the World Medical Association Declaration of Helsinki. Participants were asked for their consent and were guaranteed anonymity in the information collected. The institutional ethics board of the Umberto I teaching hospital/Sapienza University of Rome approved this study (Protocol 226/2021).

### Questionnaire

The questionnaire consisted of 27 questions grouped into three sections. It was offered in Italian or English according to the student’s preference and took approximately 5 min to fill out. The first section of the questionnaire aimed to collect socio-demographic characteristics: financial status, university registration number, age, gender, nationality, faculty, year of study, COVID-19 vaccination uptake, and the COVID-19 history (previous SARS-CoV-2 infections, symptoms, and disease outcome) of each student and their acquaintances. This section also investigated the work experience of students and their families during the pandemic (e.g., career change or job loss). The second section explored anxiety due to COVID-19 and the need for professional psychological support. The Coronavirus Anxiety Scale (CAS) (14), a validated self-reporting mental health screener of dysfunctional anxiety associated with the coronavirus crisis, was used. This five-item scale investigates dizziness, sleep disturbances, tonic immobility, appetite loss, and abdominal distress. Each item is rated on a five-point scale, from 0 (not at all) to 4 (nearly every day), based on experiences over the past 2 weeks. The CAS allows accurate discrimination (90% sensitivity and 85% specificity) between individuals with and without dysfunctional anxiety using an optimized cut-off score of ≥9 (14). The third section explored the students’ self-reported change in academic performance. Academic performance was measured as the grades received in examinations. The interviewees were asked whether during the Covid-19 pandemic their personal academic performance (i) had decreased, (ii) was unchanged, (iii) had increased or whether (iv) they were not able to make a comparison. This section also investigated how much distance learning had impacted the students’ personal and university experience, rating answers on a five-point scale from 0 (definitely not) to 4 (definitely yes).

### Statistical analysis

Descriptive statistics were obtained using mean and standard deviation, or median and interquartile range, for continuous variables and proportions for categorical and dichotomous variables. Students were considered to be either Italian or non-Italian. The faculties were divided into three distinct areas of study: Healthcare (e.g., medicine, nursing), Science & Technology (e.g., biology, mathematics, engineering), or Social Sciences & Humanities (e.g., law, economics, philosophy). Students were grouped into two categories based on their year of study: those in their first, second, or third year, and those in their fourth year or beyond. This classification was chosen because, in Italy, most university programs offer a first-level degree (bachelor’s degree) after 3 years of study. Consequently, “students in the fourth year or beyond” includes those enrolled in postgraduate programs and those in the fourth year or higher of longer programs, such as medicine or law. For the purposes of this analysis, financial status was dichotomized into two groups: Many/Some difficulties vs. Managing well enough/Managing very well. Answers relating to the need for professional psychological support were collapsed into two groups: yes (regardless of the type and timing of psychological support needed) vs. no (never felt the need). Coronavirus Anxiety Scale (CAS) scores were divided into groups using a cut-off score of ≥9: anxious disorder not present (i.e., scores <9) vs. dysfunctional anxiety due to COVID-19 (i.e., ≥ 9). Answers relating to having contracted a SARS-CoV-2 infection were collapsed into two groups: yes (i.e., asymptomatic, mild symptoms or mild or severe symptoms) vs. no (i.e., never). Those who had received at least one dose of vaccine were counted as vaccinated, the remainder as unvaccinated. Change in academic performance, our main outcome, was classified into three groups: increased or unchanged, decreased or not able to make a comparison. Survey participants in the last group, i.e., who were unable to make a comparison, were excluded from further analysis. A univariable analysis was performed to assess possible associations between each variable and the outcome, defined as a decline in academic performance. For the univariable analysis, the Wilcoxon rank-sum test was used to compare continuous variables with the outcome, whereas Pearson’s chi-squared test or Fischer’s exact test was used for dichotomous and categorical variables. A multivariable logistic regression model was built to identify predictors of decline in academic performance. According to the strategy for building the logistic regression model, we included variables according to experts’ opinion and based on what has already been reported in the literature. Due to the close correlation with the outcome and the fact that these are also consequences of the educational reorganization that occurred during the pandemic, we opted not to incorporate variables relating to the influence of distance/mixed learning in the model.

Multicollinearity was checked using as threshold a variance inflation factor of 2.5 and a tolerance of 0.5. The Hosmer and Lemeshow test, the Akaike Information Criterion (AIC) and the Bayesian Information Criterion (BIC) were used to evaluate the goodness of fit of the model. As a result, the final model consisted of the following variables: age (continuous), gender (dichotomous), nationality (dichotomous), area of study (categorical), year of study (dichotomous), finances (dichotomous), changes in financial resources (categorical), need for professional psychological support (dichotomous), CAS score (dichotomous), previous COVID-19 infection (dichotomous), acquaintances who have had SARS-CoV-2 infection (dichotomous), vaccination status (dichotomous). Adjusted odds ratios (ORs) and 95% confidence intervals (CIs) were calculated.

All analyses were performed using Stata (StataCorp LLC, 4905 Lakeway Drive, College Station, TX 322, United States), version 17.0. A two-sided *p*-value <0.05 was considered statistically significant.

## Results

### Characteristics of the sample

During the 2 weeks (February 28, 2022 to April 13, 2022) of a larger screening campaign implemented during the years 2021 and 2022, 1815 students were tested at least once for SARS-CoV-2. Out of them, 1,363 students responded to the questionnaire, resulting in an overall response rate of 75.1%. Our main goal was to investigate the factors that influenced the academic performance of college students. However, 206 participants mentioned that they were unable to compare their previous academic performance with their performance during the pandemic. Therefore, we excluded this group from our analysis, resulting in a final sample size of 1,157 students. Most of the sample was female (72.5%) and Italian (90.2%) with a mean age of 24.2 ± 3.8 years. The main area of study, with a small majority (37.3%), was the healthcare area; most participants (51.8%) reported that they managed their finances well enough and with no change (49.1%) in financial resources during the pandemic (Table 1).

**Table 1:** Student (*N* = 1,157) sociodemographic characteristics and mental health experience.

Variable		N (%)
Gender		
	Female	839 (72.5)
	Male	318 (27.5)
Age (years)		
	Mean (SD)	24.2 (3.8)
	Median (IQR)	23.3 (21.8–25.3)
Nationality		
	Italian	1,044 (90.2)
	Non-Italian	113 (9.8)
Area of study		
	Healthcare	431 (37.3)
	Science & Technology	376 (32.5)
	Social Sciences & Humanities	350 (30.2)
Year of study		
	First or second or third	577 (49.9)
	Fourth or above	580 (50.1)
Academic performance		
Declined		294 (25.4)
Previous COVID-19 infection		
	Asymptomatic	50 (4.3)
	Mild symptoms	174 (15.1)
	Moderate/severe symptoms	36 (3.1)
Vaccination status		
	No vaccine	5 (0.4)
	At least one dose	111 (9.6)
	Booster dose	1,041 (90.0)
Acquaintances who have had SARS-CoV-2 infection		
	Yes	972 (84.0)
Acquaintances deceased from SARS-CoV-2 infection		
	Yes	188 (16.3)
Family/housemates who lost their job		
	Yes	112 (9.7)
Family/housemates whose employment was interrupted or who changed job		
	Yes	347 (30.0)
Changes in financial resources		
	Decreased	411 (35.5)
	Unchanged	568 (49.1)
	Increased	17 (1.5)
	Unaware	161 (13.9)
Financial status		
	Many difficuties	49 (4.2)
	Some difficulties	329 (28.4)
	Managing well enough	599 (51.8)
	Managing very well	180 (15.6)
Need for professional psychological support		
	Never felt the need	522 (45.1)
	Yes, but I never asked for support from a professional	309 (26.7)
	Yes, I went to a psychologist	91 (7.9)
	Yes, I went to a psychotherapist	56 (4.8)
	Yes, I went to a psychiatrist	11 (1.0)
	Yes, but I was already seeing a professional for support before the pandemic	56 (4.8)
	Yes, I applied to the counseling service offered by Sapienza	14 (1.2)
	Yes, but I cannot request it now (lack of time, scarce economic resources, other)	98 (8.5)
Coronavirus Anxiety Scale (CAS) score		
	Anxiety disorder not present	1,024 (88.5)
	Dysfunctional anxiety due to COVID-19	133 (11.5)

Results are expressed as mean (standard deviation, SD), median (interquartile range, IQR), or frequency (percentage). COVID-19, coronavirus disease 2019.

### Impact on mental health

More than half (54.9%) of the sample felt the need for professional psychological support. A further 26.7% reported that they needed psychological support, but never asked for it, while 13.7% of the students started visiting a professional during the pandemic (7.9% went to a psychologist, 4.8% to a psychotherapist, and 1.0% to a psychiatrist). According to the CAS results, more than one in 10 (11.5%) students were suffering from a dysfunctional anxiety disorder due to the COVID-19 pandemic (cut-off score of ≥9; Table 1).

### Impact on academic performance

A total of 25.4% of the study participants referred to a decline in academic performance (Table 1). Univariable analysis showed some differences in socio-demographic characteristics between students who reported decreased academic performance compared to those who reported increased/unchanged academic performance: mean age (24.55% vs. 24.02%, *p* = 0.016), not being an Italian national (14% vs. 8.3%, *p* = 0.005), having many/some financial difficulties (45.2% vs. 28.4%, *p* < 0.001), and the need for professional psychological support (71.4% vs. 49.3%, *p* < 0.001) were all associated with a decline in academic performance. However, experiencing dysfunctional anxiety due to the pandemic, as assessed by the CAS score, was not meaningfully associated with outcome and, in addition, no significant difference was observed for gender, area of study, and years of study (Table 2).

**Table 2:** Student sociodemographic characteristics by academic performance, coronavirus anxiety scale and psychological impact of the pandemic.

		Academic performance		*p*-value*
		Improved or unchanged	Decreased	
Gender				0.785
	Female	624 (72.3)	215 (73.1)	
	Male	239 (27.7)	79 (26.9)	
Age (years)				0.016
	Mean	24.02 (3.73)	24.55 (3.90)	
	Median (IQR)	23.22 (21.65–25.18)	23.72 (22.00–25.93)	
Nationality				0.005
	Italian	791 (91.7)	253 (86.1)	
	Non-Italian	72 (8.3)	41 (14.0)	
Area of study				0.109
	Healthcare	335 (38.8)	96 (32.7)	
	Science & Technology	268 (31.1)	108 (36.7)	
	Social Sciences & Humanities	260 (30.1)	90 (30.6)	
Year of study (N = 1,134)ª				0.685
	First, second or third	428 (50.5)	149 (51.9)	
	Fourth or above	419 (49.5)	138 (48.1)	
Financial status				< 0.001
	Many/Some difficulties	245 (28.4)	133 (45.2)	
	Managing well enough/ Managing very well	618 (71.6)	161 (54.8)	
Need for professional psychological support				< 0.001
	Yes	425 (49.3)	210 (71.4)	
Coronavirus Anxiety Scale (CAS) score				0.127
	Anxiety disorder not present	771 (89.3)	253 (86.1)	
	Dysfunctional anxiety due to COVID-19	92 (10.7)	41 (14.0)	

Results are expressed as mean (standard deviation, SD), median (interquartile range, IQR), or frequency (percentage). ªTotal number of respondents. COVID-19, coronavirus disease 2019. *Pearson’s chi-squared test or Fisher’s exact test for categorical variables and Wilcoxon rank-sum test for continuous variables.

A greater proportion of students with a decline in academic performance reported having a family member or housemate whose employment was interrupted or who changed their job (43.5% vs. 25.4%, *p* < 0.001) and experiencing a decrease in financial resources during the COVID-19 pandemic (51.7% vs. 30%, *p* < 0.001). None of the other variables showed any meaningful association with the outcome (Table 3). Finally, Table 4 shows that a greater proportion of students who reported a decrease in the number of exams taken also reported a decline in academic performance compared to those reporting an improvement or no change (71.1% vs. 7.9%, *p* < 0.001). In all questions regarding the impact of the new hybrid teaching methods, the mean of the responses was significantly higher, showing a more negative perception in students with a decline in academic performance than in those with increased or unchanged academic performance. Specifically, it was found that distance/mixed exams negatively affected academic performance more in students who reported a decline in academic performance (mean: 2.00 vs. 0.93, *p* < 0.001). In addition, students with a decline in academic performance were more likely to report that distance/mixed learning required more study hours (mean: 2.04 vs. 1.27, *p* < 0.001) and made it more difficult to obtain insights (mean: 2.15 vs. 1.52, *p* < 0.001) and to focus during the lectures (mean: 2.90 vs. 2.15, *p* < 0.001). Finally, students with a decline in academic performance were more likely to feel that distance/mixed learning negatively affected their social relationships with other students (mean: 3.00 vs. 2.40, *p* < 0.001), their relationships with professors/university staff (2.50 vs. 1.82, *p* < 0.001) and their social relations with people not belonging to the University (mean: 2.47 vs. 1.77, *p* < 0.001).

**Table 3:** Student COVID-19 experience, vaccination status and impact of the pandemic by academic performance.

		Academic performance		*p*-value*
		Increased or unchanged	Decreased	
Previous SARS-CoV-2 infection				0.738
	No infection	667 (77.3)	230 (78.2)	
	Infection	196 (22.7)	64 (21.8)	
Vaccination status				0.624
	Unvaccinated	4 (0.5)	1 (0.3)	
	Vaccinated	859 (99.5)	293 (99.7)	
Acquaintances who have had SARS-CoV-2 infection				0.027
	Yes	713 (82.6)	259 (88.1)	
Acquaintances deceased from SARS-CoV-2 infection				0.683
	Yes	138 (16.0)	50 (17.0)	
Family/housemates who lost their job				0.004
	Yes	71 (8.2)	41 (14.0)	
Family/housemates whose employment was interrupted or who changed job				< 0.001
	Yes	219 (25.4)	128 (43.5)	
Changes in financial resources				< 0.001
	Decreased	259 (30.0)	152 (51.7)	
	Unchanged	461 (53.4)	107 (36.4)	
	Increased	13 (1.5)	4 (1.4)	
	Unaware	130 (15.1)	31 (10.5)	

Results are expressed as mean (standard deviation, SD), median (interquartile range, IQR), or frequency (percentage). COVID-19, coronavirus disease 2019. *Pearson’s chi-squared test or Fisher’s exact test for categorical variables and Wilcoxon rank-sum test for continuous variables.

**Table 4:** Impact of educational reorganization on academic performance, from 0 (definitely not) to 4 (definitely yes).

		Academic performance		*p*-value*
		Increased or unchanged	Decreased	
Number of exams				< 0.001
	Decreased	68 (7.9)	209 (71.1)	
	Unchanged	624 (72.3)	73 (24.8)	
	Increased	171 (19.8)	12 (4.1)	
Distance/mixed exams negatively affected academic performance				< 0.001
	Mean	0.93 (1.16)	2.00 (1.41)	
	Median (IQR)	0 (0–2)	2 (1–3)	
Distance/mixed learning requires more study hours				< 0.001
	Mean	1.27 (1.27)	2.04 (1.40)	
	Median (IQR)	1 (0–2)	2 (1–3)	
Difficulty to obtain insights due to distance/mixed learning				< 0.001
	Mean	1.52 (1.35)	2.15 (1.32)	
	Median (IQR)	2 (0–2)	2 (1–3)	
Distance/mixed learning made it difficult to focus during the lectures				< 0.001
	Mean	2.15 (1.48)	2.9 (1.32)	
	Median (IQR)	2 (1–3)	3 (2–4)	
Distance/mixed learning negatively affected social relationships with other students				< 0.001
	Mean	2.40 (1.39)	3.00 (1.24)	
	Median (IQR)	3 (1–4)	4 (2–4)	
Distance/mixed learning negatively affected relationships with professors/university staff				< 0.001
	Mean	1.82 (1.35)	2.50 (1.30)	
	Median (IQR)	2 (1–3)	3 (2–4)	
Distance/mixed learning negatively affected the social relations with people not belonging to the University				< 0.001
	Mean	1.77 (1.42)	2.47 (1.36)	
	Median (IQR)	2 (0–3)	3 (2–4)	

Results are expressed as mean (standard deviation, SD), median (interquartile range, IQR), or frequency (percentage). *Pearson’s chi-squared test or Fisher’s exact test for categorical variables and Wilcoxon rank-sum test for continuous variables.

### Predictors of decline in academic performance

In the multivariable analysis, higher odds of decline in academic performance were found for students who felt the need for professional psychological support (aOR: 2.58, 95% CI: 1.87–3.55; Table 5). Similarly, being non-Italian was associated with a higher likelihood of decline in academic performance (aOR: 2.12, 95% CI: 1.29–3.48). As for the area of study, only students enrolled in a science and technology faculty had higher odds of a decline in academic performance than healthcare students (aOR: 1.81, 95% CI: 1.27–2.57). On the other hand, being in a higher study year and having good or very good financial status were negatively associated with a decline in academic performance (aOR: 0.70 95% CI: 0.52–0.96, and aOR: 0.64, 95% CI: 0.47–0.88, respectively). Furthermore, students who experienced a decrease in their financial resources were more likely to experience a decline in academic performance than students who reported no financial changes or were unaware of this information. (aOR: 0.49, 95% CI: 0.35–0.67; aOR: 0.45, 95% CI: 0.28–0.73, respectively). By contrast, age, gender, previous COVID-19 infection, CAS score, infection of acquaintances and vaccination status did not seem to be predictors of the outcome (Table 5). The goodness of fit of the model was confirmed by the Hosmer and Lemeshow test (*p* = 0.7) and the values of AIC and BIC, respectively 1199.3 and 1279.8. Both VIF and tolerance values were lower than the prespecified thresholds.

**Table 5:** Multivariable logistic regression model for the decline in academic performance among the students surveyed between 28 February and 13 April 2022, Sapienza University of Rome (*N* = 1,134).

	Decline in academic performance		
	Odds ratio	95% CI	*p*-value
Age	1.03	(0.99–1.07)	0.122
Gender			
Female	Ref.		
Male	1.03	(0.73–1.44)	0.876
Nationality			
Italian	Ref.		
Non-Italian	2.12	(1.29–3.48)	0.003
Area of study			
Healthcare	Ref.		
Science & Technology	1.81	(1.27–2.57)	0.001
Social Sciences & Humanities	1.14	(0.79–1.64)	0.483
Year of study			
First, second or third	Ref.		
Fourth or above	0.70	(0.52–0.96)	0.024
Finances			
Many/some difficulties	Ref.		
Managing well enough/Very well	0.64	(0.47–0.88)	0.006
Changes in financial resources			
Decreased	Ref.		
Increased	0.84	(0.25–2.84)	0.780
Unchanged	0.49	(0.35–0.67)	0.001
Unaware	0.45	(0.28–0.73)	0.001
Previous COVID-19 infection			
No infection	Ref.		
Infection	0.89	(0.63–1.27)	0.529
Acquaintances who have had SARS-CoV-2 infection			
No infection	Ref.		
Infection	1.45	(0.95–2.22)	0.086
Vaccination status			
Unvaccinated	Ref.		
Vaccinated	1.52	(0.15–15.28)	0.721
Need for professional psychological support			
Never needed it	Ref.		
Felt the need	2.58	(1.87–3.55)	0.001
CAS Score			
Anxiety disorder not present	Ref.		
Dysfunctional anxiety due to COVID	0.97	(0.63–1.50)	0.903

CI, Confidence Interval. COVID-19, Coronavirus disease 2019.

## Discussion

This study shows that 25.4% of students who participated in Sapienza University’s SARS-CoV-2 screening campaign reported a decline in academic achievement, indicating that the pandemic affected the performance of at least some students. As previously found in one research study conducted in Italy at a university enrolled during lockdown, distance education has affected students on several fronts (10), which is why we decided to investigate student opinion of this new form of teaching imposed by the pandemic. When evaluating the effects of educational restructuring on students, two main areas emerged as being negatively impacted by the transition to hybrid or remote teaching. The first is concentration, and the second is sociability. Attentional difficulty and loss of concentration during classes conducted online are two of the disadvantages of this new form of education, alongside technical and computer difficulties and insufficient assistance for individuals with special needs (15, 16). However, students also noted some advantages of this new situation, such as continuous access to online materials and financial savings due to a reduced need to commute to the university (15–17). Unsurprisingly, the social aspect of university life was severely disrupted by the pandemic, with students reporting impoverished relationships with their peers, professors, university staff, and non-university acquaintances. The lack of pre-existing close relationships might explain why first-year students found it more difficult to sustain a good academic performance. Efforts by Sapienza University to ensure a safe return to in-person activities have been made precisely with the view that social and interpersonal relationships are an indissociable part of teaching and learning at any level and are a key factor in maintaining mental well-being.

Therefore, it was of great interest to assess how the pandemic affected the mental health of the study participants. There is much evidence that students felt overwhelmed by the changes caused by the pandemic (5), reporting more mood disorder symptoms, perceived stress, and alcohol use than pre-pandemic same year students did (18), and our results confirmed what has already been observed in the literature. An alarming finding is that more than 50% of the sample surveyed had felt the need for psychological support; while this brings to our attention and confirms the severe impact of COVID-19 on the psychological well-being of the population (19), it also underscores how students are among the most susceptible when it comes to mental health issues (20, 21), although other categories, such as resident doctors, reported similar levels of mood disorders and sleep disturbances (22). If almost 27% of those who felt the need for support did not turn to a professional we must question the actual availability and accessibility of psychological care services for younger people. On the other hand, psychological and psychiatric care services, like educational services, have undergone a forced reorganization, even though they remain of vital importance, especially in times of crisis and uncertainty such as the COVID-19 emergency (23). Remarkably, despite the fact that the survey was administered 2 years after the onset of the pandemic, the CAS results (14) on dizziness, sleep disturbance, tonic immobility, appetite loss, and abdominal distress show how far the consequences of this period, particularly those related to anxiety and psychological discomfort, go beyond the emergency phase and the outbreak of infection. The reorganization of study and workspaces, uncertainty about the future, and the disruption of social life are just some of the issues that still make it difficult for many individuals to regain and maintain their psychological well-being. This is supported by the finding that, within our sample, more than one in 10 still suffer from a dysfunctional anxiety disorder related to the pandemic. Given this evidence, despite the end of the emergency period, Sapienza University of Rome has initiated a free psychological counseling service for students coinciding with the return to in-person activities.

We assessed academic performance based on grades, as this is the practice in the literature (24). Excluding the just over 200 students who could not compare their pre- and post-pandemic grades, we analyzed the characteristics of the remaining 1,157 respondents. Among the predictor variables of a decline in performance, it is noteworthy that two relate to the financial assets of the student or their family: having a good or very good financial baseline turns out to be a protective factor among those considered, while having had a worsening financial situation during the pandemic is associated with a decline in academic performance. As might be expected, consistent with previous studies, this reinforces the notion that it is appropriate to frame the pandemic as one component of what is actually a syndemic, in which environmental, social and economic problems exacerbate the consequences of health problems, in this case represented by the widespread occurrence of SARS-CoV-2 infections (25) This is all the truer for the student population, for whom economic difficulties have increased, both for those who rely on family support and for those who work (6). Many students were forced to suspend or drop out of their studies, contributing to the already problematic deterioration of their mental health (7, 8). Having felt the need for psychological assistance is among the predictors of decline and points to how mental health and academic performance are closely and inextricably linked (17): a psychological distress as unexpected and prolonged as that caused by the pandemic has impacted the students’ daily lives as much as their academic lives. With regard to student characteristics, it is clear how already being in college for several years, and consequently having already developed a method of study, relationships with colleagues and professors, and having already had experience of examination methods, decreases the likelihood of worsening achievement during the pandemic. Some subject areas also require more hands-on practice than others, for example, laboratory activities or internships, and this is perhaps why students in science and technology faculties were found to be more prone to a decline in performance during the pandemic, a period when in-person activities were forcibly discontinued or severely limited and reorganized. Prior evidence shows that being a foreign student increases the difficulty of performing well in college, and according to our analysis model, the pandemic only exacerbated this situation. Previous research (26) has provided evidence suggesting that being a foreign student poses additional challenges when it comes to achieving academic success in college. During the pandemic, foreign students lost scholarships and casual employment that they usually used to support themselves during their studies, resulting in the need for urgent material as well as, being away from their families, emotional support (27). Our analysis model, in line with this prior evidence, indicates that COVID-19 has further intensified challenges in minimizing the decline in academic performance for this specific student population. Previous SARS-CoV-2 infection, on the other hand, does not appear to be linked to a change in academic performance. This could potentially suggest that the impact on academic performance was largely due to the consequences and changes imposed by the pandemic and not by the infectious event itself.

This study has some limitations. First, the cross-sectional design prevented us from drawing causal conclusions between the decline in academic performance and associated factors. Second, since participants in this study were recruited from those participating in the SARS-CoV-2 screening campaign, it is possible that, despite the good response rate obtained, our sample may not be fully representative of Sapienza University students. Moreover, because respondents were asked to fill out the questionnaire while waiting to be tested, the survey was limited to a small number of questions, reducing the scope of the survey. Despite this, we were able to analyze the impact of educational reorganization on students, the influence of the pandemic on mental health, students’ personal and family experience of COVID-19, and factors associated with changes in academic achievement, providing data that should be invaluable for policymakers responsible for reorganizing educational institutions, for maintaining psychological well-being and for avoiding a widespread decline in academic performance. Future crises may indeed disrupt education again and impact students’ well-being. Based on the results of our research, we believe that universities should develop emergency plans aimed at ensuring a robust support system for academic needs in order to minimize the proportion of students who, as we have found, may experience a decline in performance. Our survey confirmed the significant impact that the pandemic has had on mental health, and for this reason, emergency plans should certainly include psychological support interventions. The creation of resilient infrastructures and the promotion of strong online communities could mitigate the negative effects of disruptions to in-person teaching, disruptions which, as emerged, were perceived even more negatively by those who reported a decline in academic performance. These findings highlight the necessity for preparedness and resilience planning in higher education to better manage future crises.

## Conclusion

Having a complete picture of not only the academic, but also the wider, factors that influence student performance, such as anxiety and other mental health issues (28), is crucial if policymakers are to intervene to ensure student success despite the challenges posed by the pandemic. The perceived need for psychological support in the aftermath of the COVID-19 emergency appears to have had an impact on the decline in academic performance and mental health of students. Therefore, there is the need for universities to develop contingency plans and resilient infrastructures to ensure robust support systems for both academic and mental health needs to better handle future crises. In addition, we found that the self-reported decline in academic performance was associated with the economic situation of individual students and their households, suggesting that inequality has exacerbated the negative effects of the COVID-19 emergency on this subpopulation.

## Data availability statement

The raw data supporting the conclusions of this article will be made available by the authors, without undue reservation.

## Ethics statement

The studies involving human participants were reviewed and approved by the Institutional Ethics Board of the Policlinico Umberto I Teaching Hospital/Sapienza University of Rome (protocol number 226/2021). The patients/participants provided their written informed consent to participate in this study.

## Author contributions

LS: Conceptualization, Data curation, Formal analysis, Writing – original draft. IM: Writing – original draft. ER: Conceptualization, Investigation, Writing – original draft. VB: Conceptualization, Data curation, Formal analysis, Writing – review & editing. GM: Data curation, Formal analysis, Writing – review & editing. AS: Investigation, Writing – review & editing. AC: Investigation, Writing – review & editing. CV: Writing – review & editing. CM: Writing – review & editing. PV: Writing – review & editing. AM: Data curation, Supervision, Writing – original draft.
