# Supplementary material for: Decline in academic performance and mental health during the COVID-19 pandemic: a cross-sectional survey among Sapienza University of Rome students
Source: Front Public Health. 2024 Jul 17;12:1408191. doi: 10.3389/fpubh.2024.1408191 (PMC11288869; doi:10.3389/fpubh.2024.1408191)
Supplement: Supplementary file 1 [file Table_1.DOCX]

|  | age | gender | nationality | area of study | year of study | finances | changes in fin resources | need for professional psychological support | CAS Score | previous COVID-19 infection | acquaintances who have had SARS-CoV-2 infection | vaccination status |
| --- | --- | --- | --- | --- | --- | --- | --- | --- | --- | --- | --- | --- |
| age | 1.0000 |  |  |  |  |  |  |  |  |  |  |  |
| gender | 0.0890 | 1.0000 |  |  |  |  |  |  |  |  |  |  |
| nationality | 0.2239 | 0.0366 | 1.0000 |  |  |  |  |  |  |  |  |  |
| area of study | -0.0255 | 0.0432 | -0.0396 | 1.0000 |  |  |  |  |  |  |  |  |
| year of study | 0.3356 | 0.0286 | 0.0913 | 0.1135 | 1.0000 |  |  |  |  |  |  |  |
| finances | -0.1624 | -0.0050 | -0.0679 | -0.0690 | -0.0499 | 1.0000 |  |  |  |  |  |  |
| changes in fin resources | -0.0757 | 0.0393 | -0.0089 | -0.0368 | -0.0485 | 0.3129 | 1.0000 |  |  |  |  |  |
| need for professional psychological support | -0.0462 | -0.1963 | -0.0855 | 0.0999 | 0.0461 | -0.0948 | -0.1467 | 1.0000 |  |  |  |  |
| CAS Score | 0.0279 | -0.0917 | 0.0137 | 0.0737 | 0.0426 | -0.0570 | -0.0604 | 0.2193 | 1.0000 |  |  |  |
| previous COVID-19 infection | 0.0150 | -0.0006 | 0.0619 | -0.0397 | -0.0672 | 0.0117 | 0.0263 | -0.0290 | -0.0202 | 1.0000 |  |  |
| acquaintances who have had SARS-CoV-2 infection | -0.0066 | -0.0284 | -0.1048 | 0.0055 | 0.0141 | 0.0031 | -0.0767 | 0.1004 | -0.0303 | 0.0552 | 1.0000 |  |
| vaccination status | -0.0170 | 0.0108 | -0.0279 | 0.0611 | -0.0145 | 0.0389 | 0.0481 | 0.0202 | -0.0186 | -0.0609 | 0.0442 | 1.0000 |

**Table S1.** Correlation matrices of the model variables.
